# Tick-Borne Encephalitis Virus Structural Proteins Are the Primary Viral Determinants of Non-Viraemic Transmission between Ticks whereas Non-Structural Proteins Affect Cytotoxicity

**DOI:** 10.1371/journal.pone.0158105

**Published:** 2016-06-24

**Authors:** Maxim A. Khasnatinov, Andrew Tuplin, Dmitri J. Gritsun, Mirko Slovak, Maria Kazimirova, Martina Lickova, Sabina Havlikova, Boris Klempa, Milan Labuda, Ernest A. Gould, Tamara S. Gritsun

**Affiliations:** 1 Federal State Public Science Institution «Scientific Centre of Family Health and Human Reproduction Problems», Irkutsk, Russian Federation; 2 School of Molecular and Cellular Biology and Astbury Centre for Structural and Molecular Biology, Faculty of Biological Sciences, University of Leeds, Leeds, United Kingdom; 3 Oxford Progress Ltd, Oxford, United Kingdom; 4 Institute of Zoology, Slovak Academy of Sciences, Bratislava, Slovakia; 5 Institute of Virology, Slovak Academy of Sciences, Bratislava, Slovakia; 6 Charité School of Medicine, Institute of Virology, Berlin, Germany; 7 Faculté de Médecine, UMR 190 "Emergence des Pathologies Virales", Aix Marseille Université, Marseille, France; University of Maryland, College Park, UNITED STATES

## Abstract

Over 50 million humans live in areas of potential exposure to tick-borne encephalitis virus (TBEV). The disease exhibits an estimated 16,000 cases recorded annually over 30 European and Asian countries. Conventionally, TBEV transmission to *Ixodes* spp. ticks occurs whilst feeding on viraemic animals. However, an alternative mechanism of non-viraemic transmission (NVT) between infected and uninfected ticks co-feeding on the same transmission-competent host, has also been demonstrated. Here, using laboratory-bred *I*. *ricinus* ticks, we demonstrate low and high efficiency NVT for TBEV strains Vasilchenko (Vs) and Hypr, respectively. These virus strains share high sequence similarity but are classified as two TBEV subtypes. The Vs strain is a Siberian subtype, naturally associated with *I*. *persulcatus* ticks whilst the Hypr strain is a European subtype, transmitted by *I*. *ricinus* ticks. In mammalian cell culture (porcine kidney cell line PS), Vs and Hypr induce low and high cytopathic effects (cpe), respectively. Using reverse genetics, we engineered a range of viable Vs/Hypr chimaeric strains, with substituted genes. No significant differences in replication rate were detected between wild-type and chimaeric viruses in cell culture. However, the chimaeric strain Vs[Hypr str] (Hypr structural and Vs non-structural genomic regions) demonstrated high efficiency NVT in *I*. *ricinus* whereas the counterpart Hypr[Vs str] was not transmitted by NVT, indicating that the virion structural proteins largely determine TBEV NVT transmission efficiency between ticks. In contrast, in cell culture, the extent of cpe was largely determined by the non-structural region of the TBEV genome. Chimaeras with Hypr non-structural genes were more cytotoxic for PS cells when compared with Vs genome-based chimaeras.

## Introduction

*Tick-borne encephalitis virus* (TBEV) is recognised as a re-emerging human pathogen with an estimated 16,000 cases recorded annually over 30 European and Asian countries [1, 2, 3]**.** It is currently classified as a virus species in the tick-borne flavivirus ecological group within the genus *Flavivirus* (family *Flaviviridae*) which includes three other ecological groups, *viz* mosquito-borne, no-known vector and insect-specific flaviviruses [4, 5]. The flavivirus genome is a single-stranded RNA molecule of positive polarity approximately 11kb long that encodes three structural (C, M and E) and eight non-structural (NS1, NS2A, NS2B, NS3, NS4A, 2K, NS4B and NS5) proteins. The virions are 50 nm spherical particles formed by an outer lipid coat containing membrane (M) and envelope (E) glycoproteins. Capsid proteins (C) encapsulate the viral RNA genome within an inner spherical 30 nm capsid. [6]. Envelope glycoproteins play a key role during essential stages of the virus reproductive cycle including adsorption, pH-dependent fusion and assembly [7]. Non-structural (NS) proteins are multifunctional and exhibit various activities related to assembly and functioning of the virus replication complex. The single open reading frame (ORF) of the flavivirus RNA genome is flanked by 5' and 3' untranslated regions (5' and 3'UTRs), which—along with adjacent regions of the ORF—contain essential RNA structural elements. These RNA structures are associated with promoter and enhancer functions of viral RNA replication and also play a role in suppression of cell innate immunity [6, 8–10].

TBEV circulation in the natural environment occurs via at least three different modes of transmission. Conventionally, viraemic animals inhabiting tick-infested areas serve as a source of virus for ticks when they feed on these animals. The ticks then reproduce the virus, which reaches the salivary glands and is then transmitted to a naive vertebrate host when the tick takes its second blood meal [1, 11, 12]. An alternative form of virus transmission between ticks and animals occurs when ticks co-feed on either susceptible or insusceptible animals [13, 14]. This process is defined as non-viraemic transmission (NVT) because the virus is able to infect non-infected ticks that co-feed with infected ticks on the same animal. This transmission mechanism is potentially much more efficient than viraemic transmission because it does not require the development of viraemia in the vertebrate host and the virus can be transmitted directly between the ticks even when vertebrate hosts are known to be immune to infection [13–15]. Dendritic cells at the local skin site, where co-feeding takes place, have been demonstrated as the vehicle for TBEV during this tick-to tick transmission process [14]. Finally, although a comparatively inefficient mechanism, vertical transmission of the virus from infected female ticks to the eggs and further to the larval stage has been observed [11, 12, 16].

There are a wide variety of TBEV strains, which are currently subdivided into European (EU), Siberian (SIB) and Far Eastern (FE) subtypes [4, 17, 18] that are genetically very closely related (96% amino acid similarity). In nature SIB-TBEV and FE-TBEV are associated with the Taiga tick *I*. *persulcatus* whereas EU-TBEV is typically associated with the sheep tick *I*. *ricinus*. Genetic adaptation of TBEV to these two tick species is reflected in phylogenetic trees which exhibit two distinct TBEV clades: one containing SIB-TBEV and FE-TBEV which are associated with *I*. *persulcatus* and the other containing EU-TBEV which is associated with *I*. *ricinus*. [19–23]. SIB-TBEV and FE-TBEV are the predominant subtypes in Siberia and Far Eastern Asia, where *I*. *persulcatus* prevails whilst *I*. *ricinus* is the only tick species in Western Europe that is recognised as a significant vector of EU-TBEV. In Eastern Europe, the habitats of *I*. *ricinus* and *I*. *persulcatus* are believed to have overlapped for at least 5000–6000 years [24] and SIB-TBEV and EU-TBEV have co-circulated for an estimated 1300 years [25, 26]. In spite of this, the introduction of TBEV into non-competent ticks is restricted by as yet undefined biological mechanisms that prevent long-term establishment of TBEV in ecosystems lacking highly competent ticks. Thus the distinct Baltic lineage [27], SIB-TBEV, has still only been isolated from *I*. *persulcatus* and no adaptation to *I*. *ricinus* has been demonstrated [23]. A similar situation was recorded for FE-TBEV in Europe and the Ural region of Russia, when this subtype was introduced with animals transported from far-eastern Russia during the 1930s. Later analysis demonstrated that FE-TBEV introduced in the 1930s did not establish stable foci and has only been sporadically isolated from *I*. *persulcatus* in this region of Eastern Europe and Baltic countries [21, 28]. For the first time, in the current study we define the molecular barriers that restrict TBEV adaptation to non-competent ticks.

In preliminary investigations we observed low non-viraemic transmission (NVT) rate between co-feeding *I*. *ricinus* ticks for the SIB-TBEV strain Vs in contrast to the EU-TBEV strain Hypr that demonstrated high levels of NVT. This implied the existence of a potential TBEV-tick “transmission barrier” specific for particular TBEV subtypes. To identify the mechanism of this host specificity we constructed a range of chimaeric viruses, with various combinations of Vs and Hypr genes and compared their properties in both mammalian cell culture and in co-feeding *I*. *ricinus* ticks transmission experiments. Here we demonstrate that the structural genes of Hypr virus, acting in accord, appear to determine the high NVT rate of this virus between co-feeding ticks during the NVT process. In contrast, the region of the TBEV genome encoding the non-structural proteins was responsible for differences in cytopathic activity between Vs and Hypr.

## Results

### High *I*. *ricinus* tick transmission rate for Hypr and low rate for Vs

**Fig 1:**
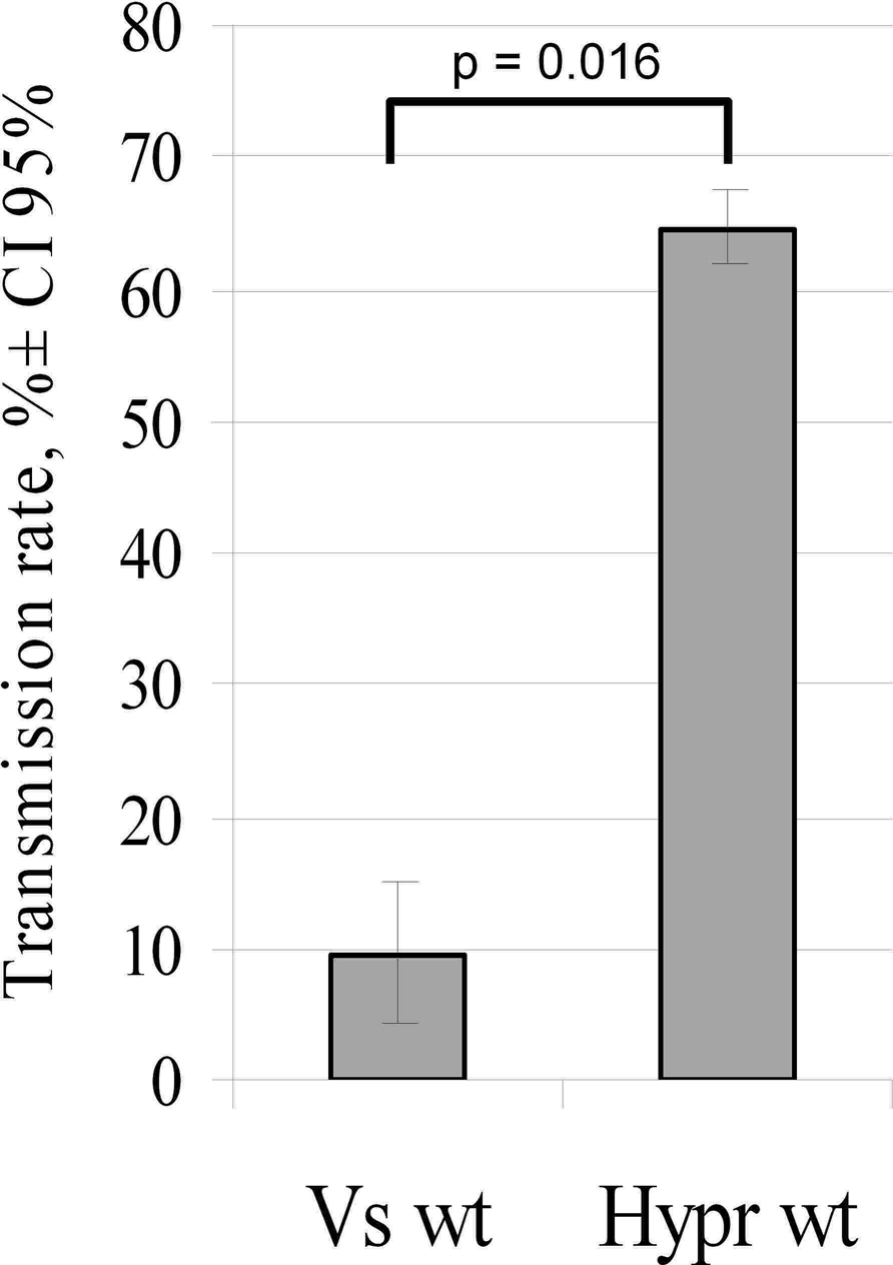
Efficiency of non-viraemic transmission of SIB-TBEV (Vs) and EU-TBEV (Hypr) between co-feeding *I*. *ricinus*. Two infected female ticks were placed in close proximity to 15 uninfected nymphs on the same skin site of laboratory mice. Following three days of co-feeding, nymphs were removed and virus titres determined by plaque titration. The mean transmission rate is expressed as percentage of positive nymphs in which TBEV was detected.

In repeated experiments, Hypr virus demonstrated a mean NVT rate of ~65% was reproduced whereas the NVT rate of Vs virus was between 5–15% i.e. significantly lower (p = 0.016) (Fig 1). The *I*. *ricinus* ticks used in these experiments are the natural vector for the EU-TBEV subtype, to which the Hypr strain belongs. Consequently we questioned whether or not the decreased NVT rate of the Vs strain was the result of adaptation to another vector tick species—in this case, *I*. *persulcatus*. To identify the genes responsible for host specificity of TBEV we replaced the genes in the Hypr genome with the homologous genes from the Vs genome and *vice versa* and tested the derived chimaeras for replication in cell culture, virulence for mice and NVT efficiency in ticks.

### Description of chimaeric viruses constructed for this investigation

**Fig 2:**
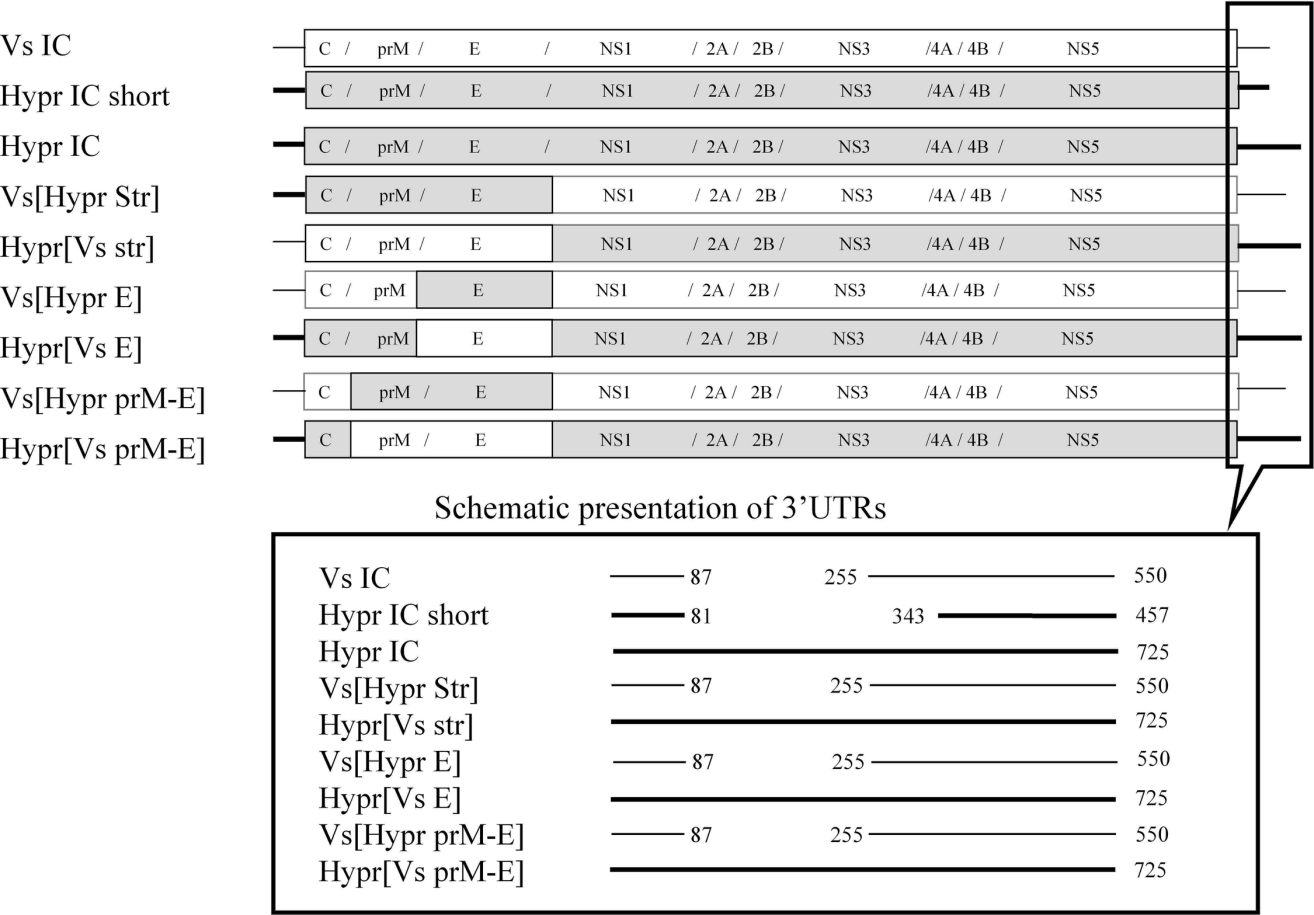
Vs and Hypr chimaeric viruses. A) The polyproteins, with individual proteins of Hypr virus (grey bars) and Vs virus (white bars) are flanked with 3`UTR (thick and thin lines for Hypr and Vs respectively). B) Schematic representation of the 3`UTRs., Numbers specify nucleotide positions in 3’UTRs following the stop codon. Internal deletions present in the Hypr and Vs genomes are identified by the numbers corresponding to those of the Hypr-long 3’UTR. Intermediate plasmids, constructed to recover Hypr, Vs and chimaeric Hypr/Vs infectious virus, are presented in S1 Fig.

Previously, structural proteins were shown to play a significant role in adaptation of TBEV to the tick host and the NVT process [29–31]. In order to investigate this further, we constructed two sets of chimaeric TBEVs (Fig 2). In one set we sequentially replaced the structural genes of Hypr virus with the equivalent genes of Vs virus. In the second set we replaced the structural genes of Vs virus with the equivalent genes of Hypr virus. Thus, we prepared three chimaeric viruses for each set with one of the following structural fragments being substituted E, prM-E or 5`UTR-C-prM-E equivalent. The construction of chimaeric viruses Vs[Hypr C] and Hypr[Vs C] was also performed. However, although multiple replications of the experiment were performed, no infectious virus was recovered for the Hypr [Vs C] chimaera containing the 5`UTR and C gene of Vs with the remainder of the genome derived from Hypr. Therefore this virus and the reciprocal Vs[Hypr C] chimaera were excluded from further experiments. As controls for these experiments infectious clones of Hypr (Hypr IC) and Vs virus (Vs IC) were used, excluding potential effects due to genetic manipulation (see Fig 2).

The 3`UTR of Hypr is shorter than in some freshly isolated TBEV strains of the three different subtypes (Fig 2). It was suggested that shortening of the Hypr TBEV 3'UTR, by deleting the internal highly variable region occurred as the result of numerous virus passages in mice and cell culture [32, 33]. To control for the effect of 3`UTR alterations, in addition to the HyprIC with a shortened 3'UTR, a Hypr-based chimaeric virus with an elongated 3`UTR was prepared.

### Non-structural genes determine TBEV cytopathogenicity

**Fig 3:**
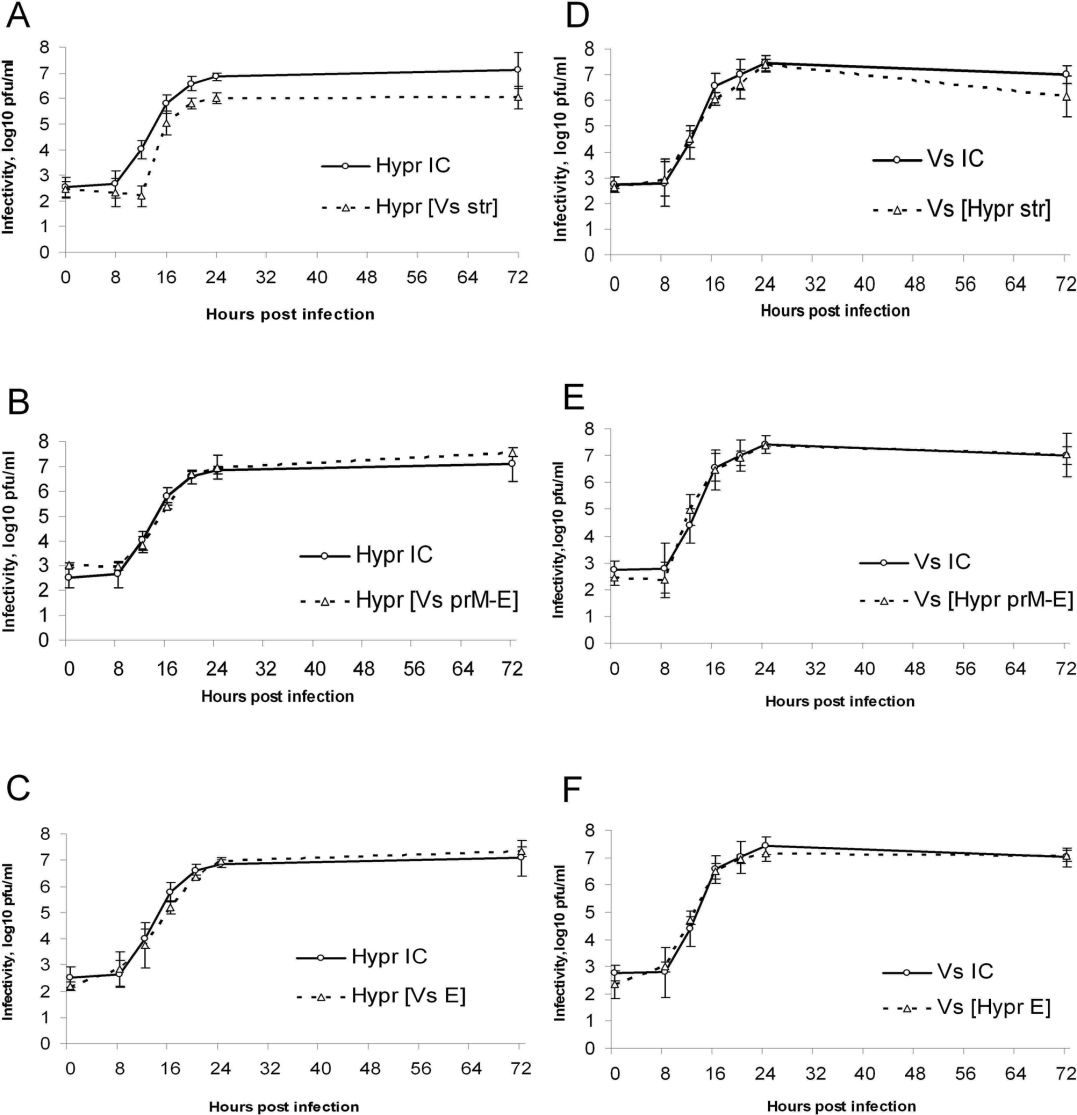
Chimaeric virus growth kinetics in cell culture. Viruses are specified as shown in the text. PS cells were infected with TBEV at a multiplicity of 1 pfu per cell in at least 4 replicates. Samples of cell culture media were collected every 4 hours during the first day pi and then once a day. Virus infectivity was established by plaque assay. A-C) HyprIC and Hypr-based recombinant viruses; D-F) VsIC and Vs-based recombinant viruses. Error bars reflect 95% confidence intervals.

To compare the viability and replication efficiency of the chimaeric viruses the growth cycle assays in porcine kidney cell cultures (PS) were carried out. All chimaeric viruses replicated efficiently and the kinetics of replication were comparable with the control viruses HyprIC and VsIC. However, the replacement of structural genes (plus the 5' UTR) in the HyprIC genome by the equivalent genes of VsIC, reduced virus infectivity by approximately one order of magnitude at each time point (Fig 3A). Interestingly, replacement of the Vs E protein alone or in combination with the Vs prM gene did not significantly affect the replication kinetics of Hypr-based chimaeras (Fig 3B and 3C). No significant differences in virus infectivity were observed between Vs based chimaeras and control VsIC, including viruses both with entire structural genes plus the 5’ UTR from Hypr and separate genes E or prM-E from HyprIC (Fig 3D–3F). Moreover, no differences in replication kinetics were revealed between HyprIC with long or short 3'UTR.

**Fig 4:**
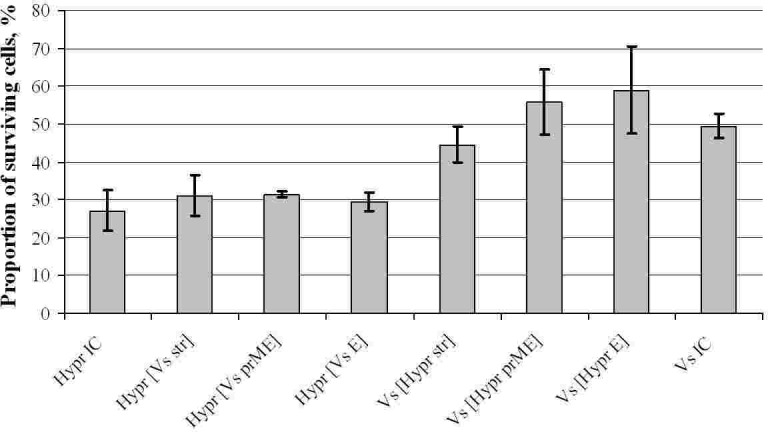
TBEV cytopathic effect in porcine kidney cells. Confluent monolayers of PS cells were infected at an moi of 1, in at least 4 replicates and incubated for 96 hours followed by staining with crystal violet and quantification of viable cells (grey blocks) as described in Materials and methods. Error bars reflect 95% confidence intervals.

Control viruses VsIC and HyprIC exhibited different intensities of cytopathic effect (cpe) in PS cells, with Hypr showing the highest and Vs the lowest cytopathic activity (Fig 4). Equivalent differences were also observed for the wild-type Vs and Hypr viruses used for the construction of VsIC and HyprIC (data not shown). Hypr based chimaeric viruses exhibited no significant differences in cytopathic activity from the HyprIC virus, all destroyed ~ 70% of the cells. Correspondingly, all chimaeric viruses with the non-structural regions of Vs virus (ie Vs[Hypr str], Vs[Hypr E] and Vs[Hypr prM-E]) were less cytopathic inducing only 30–55% of cell destruction by 96 hpi. The differences between two groups were statistically significant (p = 0.002). In conclusion, the experiments with the chimaeric Vs/Hypr viruses suggested that the level of cytopathogenicity in PS cells was largely determined by the non-structural region of the virus genome.

### Stability of chimaeric virions

**Fig 5:**
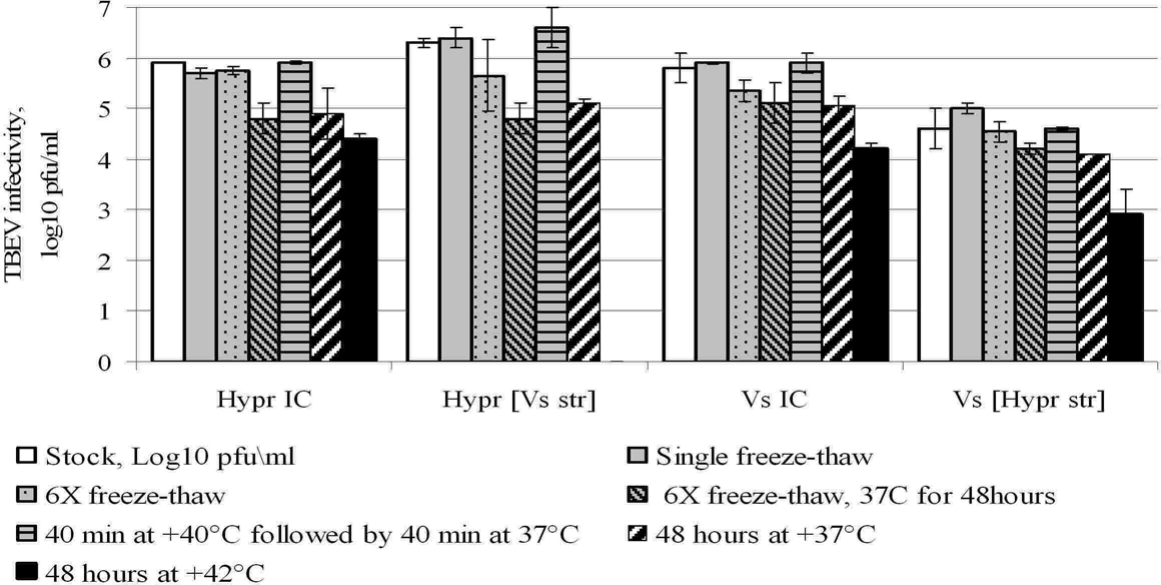
Virion stability. Identical aliquots of virus suspension were treated as indicated in chart legend (see also Materials and Methods) with virus titres determined by plaque infectivity assay. Error bars show 95% confidence intervals from the mean across three replicates.

We previously reported that Vs virions appeared to be significantly more labile when compared with other prototype TBEV strains [34] and therefore might be more sensitive to deleterious conditions during experimental procedures. The reduced yields of Hypr[Vs str] suggested the possibility that less stable virions might be formed as the result of interaction between structural proteins of Vs with Hypr genomic RNA. Accordingly, we evaluated whether or not adverse physical conditions might reduce the infectivity of the chimaeric viruses (Fig 5).

A single cycle of freezing and thawing had no significant affect on the virus titres. However, six sequential cycles of freezing and thawing caused a remarkable decrease in infectivity of Hypr[Vs str] by approximately 1 log_10_ pfu\ml though with low statistical significance (p = 0.07). A slight drop of infectivity recorded for control virus VsIC was also not statistically significant (p > 0.05). The infectivity of HyprIC and Vs[Hypr str] viruses was not affected by multiple freeze-thawing cycles. Incubation at 37°C for 48 hours significantly reduced the titres of all viruses and this was also the case after multiple freeze-thawing cycles followed by incubation at 37°C for 48 hours. The infectivity of all viruses was stable when incubated at 40°C for 40 minutes, followed by 37°C for 40 minutes. However, incubation at 42°C for 48 hours caused a significant decrease of infectivity across all viruses and indeed, Hypr[Vs str] was totally inactivated under these conditions.

In conclusion, under increased stress conditions, the Hypr[Vs str] chimaera was more labile than the other viruses possibly indicating reduced virion integrity. However, all the viruses were sufficiently stable when subjected to relatively mild physical conditions corresponding to the experimental procedures. Thus, virion lability is unlikely to have been responsible for the observed decrease of replication efficiency of Hypr[Vs str] (refer to Fig 3A).

### Non-viraemic transmission of TBEV between co-feeding ticks is primarily determined by the structural genes

**Fig 6:**
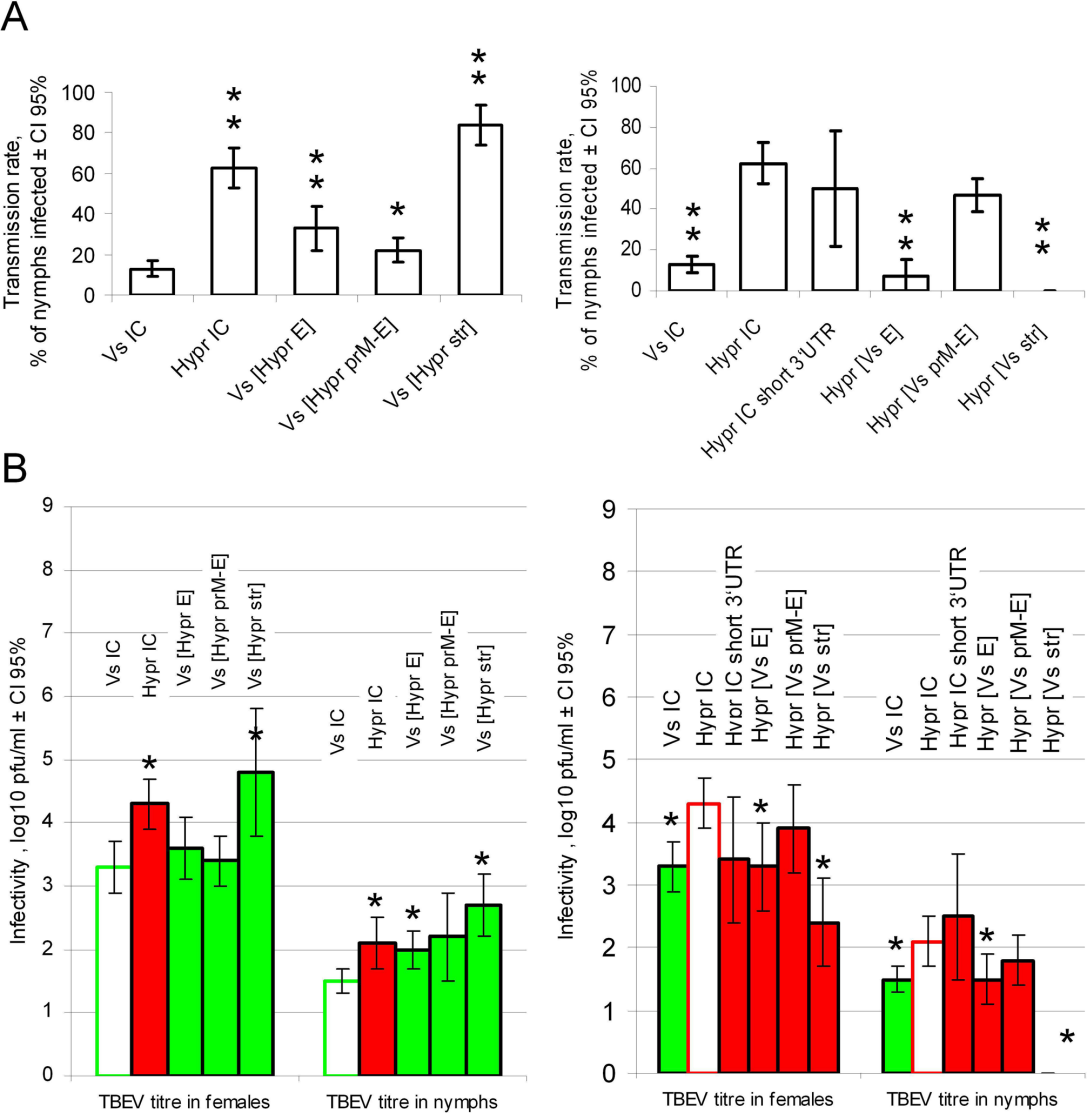
TBEV transmission and replication in *I*. *ricinus* ticks. Each adult tick female was injected with 500 pfu of virus (as specified). After 14 days two adult females were co-fed with 15 nymphs for three days and virus titres in females and nymphs were determined by plaque infectivity assay. Left and right panels illustrate biological effects of gene replacements in Vs-based and Hypr-based chimaeras respectively. Mean values are shown with error bars indicating 95% confidence intervals. A) Transmission rate of TBEV between co-feeding ticks is presented as a proportion of nymphs that acquired TBEV infection after co-feeding with infected female ticks. Depending on levels of statistically significant differences (Mann-Whitney test) between chimaeric and control viruses, results are labelled with double (p < 0.01) or single asterisks (p < 0.05). B) Virus titres of TBEV in nymphs and females of *I*. *ricinus*. Vs- and Hypr-based chimaeras are identified as green and red bars respectively; the control viruses are shown as empty bars. Highly significant differences (p < 0.05, unpaired two-tailed t-test) between chimaeric and control viruses are labelled with an asterisk.

The relative contribution of different structural and non-structural regions of the TBEV to NVT was measured for chimaeric viruses following tick co-feeding. Briefly, following co-feeding between two TBEV infected females and 15 uninfected nymphs, the female ticks and nymphs were individually tested for the presence of the virus. NVT rate was evaluated as percentage of nymphs positive for TBEV (Fig 6A). Highly significant differences between NVT rate of control recombinant viruses HyprIC and VsIC were observed (p < 0.01), which corresponded extremely closely with the NVT rates determined for the wild type Hypr and Vs viruses (Fig 1).

The most remarkable result was the increased NVT rate of chimaeric virus Vs[Hypr str] (~ 85%) which was even higher than that of HyprIC virus (~ 65%) (p = 0.002). Surprisingly, no transmission was detected for Hypr[Vs str] at all (Fig 6A). Thus, NVT efficiency is largely determined by the structural region of the TBEV genome (5'UTR-C-E-prM-E gene cluster). To investigate the contribution of individual genes and the 5’UTR, chimaeras with either exchanged E proteins alone or in combination with prM protein were constructed and transmission efficiency measured. The chimaeric viruses Vs[Hypr E] and Vs[Hypr prM-E] showed significantly higher NVT rates than control VsIC virus (Fig 6A). This implies an upregulating role for the Hypr E protein in terms of NVT. However, although significant, upregulation of NVT rate with either E protein alone or in combination with prM protein was relatively low (~ 10–20%) when compared with the upregulation of the entire 5’UTR-C-E-prM-E gene cluster (~ 80%) in Vs[Hypr str]. Therefore the 5'UTR-C genomic region of Hypr virus is an important driver of NVT and, probably, structural integrity of all virion proteins is essential for the most efficient virus NVT rate from infected females to uninfected nymphs. This conclusion is corroborated by the observed NVT rate of the chimaeras in which Vs E protein, alone or in combination with prM was engineered into the Hypr genome. The chimaeras Hypr[Vs E] and Hypr[Vs prM-E] displayed a loss of transmission efficiency (88% and 13% respectively) compared with the control HyprIC virus used for their construction. NVT rates of recombinant HyprIC and HyprIC[short 3'UTR] did not significantly differ and were close to those observed for the original parent Hypr virus (Figs 2 and 6A).

### TBEV replication in adult ticks is upregulated by viral structural genes

Following co-feeding assay, we determined TBEV infectious titres in salivary glands of the females at the end of the co-feeding period. In concordance with different NVT rates for Vs and Hypr viruses, the titre of recovered infectious virus in females differed significantly depending on which control virus was being titrated (mean 3.3 log_10_ pfu/ml for VsIC and 4.3 log_10_ pfu/ml for HyprIC, p = 0.001) (Fig 6B). Furthermore, the highest titre (~ 5 log_10_ pfu/ml) in adult ticks was recorded for chimaera Vs[Hypr str] which also showed the highest overall NVT rate. This supports the concept that the structural genes of TBEV largely determine the differences in TBEV replication efficiency, as observed between Vs and Hypr TBEV in adult ticks. Correspondingly, engineering Hypr E and prM-E genes into the Vs genome also caused an increase in Vs[Hypr E] and Vs[Hypr prM-E] titres in adult ticks when compared with the control VsIC virus. The increment was very small (p > 0.05) but was in concordance with increased NVT rates of Vs[Hypr E] and Vs[Hypr prM-E] when compared with VsIC (Fig 6A and 6B).

**Fig 7:**
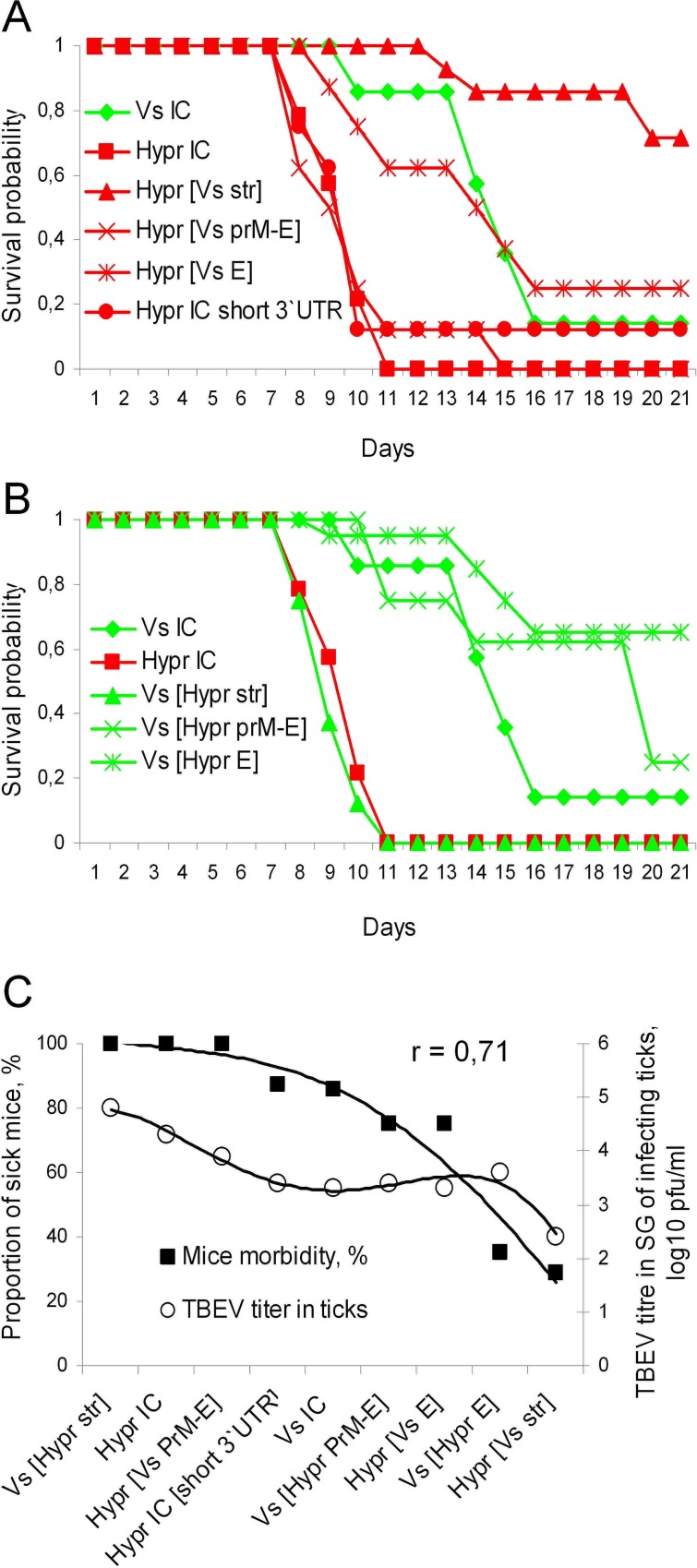
TBEV replication in mice following transmission from ticks. Mice infected via tick bites during the NVT experiments were observed for 21 days and cumulative numbers of challenged and moribund mice were analysed. A) Survival dynamics of mice infected with Vs-based (green lines/bars) chimaeras; B) Survival dynamics of mice infected with Hypr-based (red lines/bars) chimaeras. The probabilities of survival were estimated using Kaplan-Meier method; C) Correlation between mouse morbidity and infectious dose of TBEV in adult tick salivary glands following the termination of co-feeding. The data are arranged in descending order of mouse morbidity. The trend lines were drawn using the order 4 polynomial method (R^2^ ≥ 0.95). The (r) value in the top right corner of the chart reflects the Pearson’s coefficient of correlation between datasets.

Substitution of the entire VsIC 5`UTR-C-prM-E gene cluster into the Hypr genome (Hypr[Vs str]) significantly (p = 0.0002) down-regulated the TBEV replication in adult ticks by ~ 2 orders of magnitude, compared with control HyprIC virus (Fig 6B). The replacement of Hypr prM-E and E genes with those of VsIC also caused a reduction of virus titres in adult ticks in relation to control HyprIC virus although to much less extent. Average titres for Hypr[Vs prM-E] comprised 3.9 pfu/ml and difference with control HyprIC virus was not significant (p > 0.05), whereas Hypr[Vs E] exhibited a significantly reduced (p = 0.03) titre of 3.3 log_10_ pfu/ml compared to 4.3 log_10_ pfu/ml for HyprIC (Fig 7A). Introduction of the shorter 3`UTR of Hypr virus caused a remarkable decrease of viral reproduction in adult ticks with average titres for HyprIC and HyprIC [short 3'UTR] of 4.3 and 3.4 log_10_ pfu/ml respectively, however, this was not statistically significant (p > 0.05).

Thus, the differences in TBEV replication in adult ticks between most chimaeric TBEV strains as well as Hypr and Vs did not exceed 1 log_10_ pfu/ml, with the exception of Hypr[Vs str], which exhibited reduced reproduction in adult ticks by ~2 log_10_ pfu/ml compared with control HyprIC virus (with the longer 3'UTR). Control HyprIC and chimaeric Vs[Hypr str] viruses generated the highest infectious titres in *I*. *ricinus* adult ticks whilst Hypr[Vs str] virus generated the lowest titres. The other chimaeric viruses exhibited a range of intermediate virus titres, with insignificant differences in repeated experiments (at least eight females were analyzed for each virus). Nevertheless, these small yet reproducible differences could have an impact on overall virus biological characteristics [35–37].

### TBEV replication in nymphs is upregulated by TBEV structural genes

Levels of TBEV infectivity in nymphs reflect virus accumulation and reproduction during tree days of co-feeding with infected female ticks (Fig 6B). With the exception of HyprIC short (which has very large variability of titres) the differences in virus replication in nymphs between the recombinant viruses correlated with results in adult ticks (Fig 6A). The mean titres generated in nymphs by the control viruses were 2.1 log_10_ pfu/ml for HyprIC and 1.5 log_10_ pfu/ml for VsIC (Fig 6B). The highest titres (2.7 log_10_ pfu/ml) were observed for Vs[Hypr str], which again is in concordance with results in adult ticks.

The VsIC-based viruses, with substituted Hypr E and prM-E genes, exhibited increased infectivity in comparison to control VsIC virus—although for Vs [Hypr prM-E], the differences were not statistically significant (p > 0.05). In correlation with this, the HyprIC based chimaeras with E and prM-E of VsIC virus had lower infectivities in comparison with HyprIC but for Hypr [Vs prM-E] this was also not statistically significant. Similarly, as with the NVT data, no infectious Hypr[Vs str] virus was recovered from nymphs following co-feeding with infected adults (Fig 6A). The infectious titre of HyprIC [short 3'UTR] comprised 2.5 log_10_ pfu/ml and was not significantly different from HyprIC (p > 0.05).

### Structural genes determine TBEV pathogenicity in mouse model

All chimaeric viruses were pathogenic for the Balb/C mice that were used for modeling NVT of TBEV between co-feeding ticks (Fig 7, Table 1). The control viruses HyprIC and VsIC viruses exhibited reproducible differences in animal survival dynamics (average survival time (AST) of 10 dpi and 15 dpi respectively), though the difference in morbidity rates was not significant (100% and 86% respectively, p > 0.05). In agreement with earlier results, introduction of the Vs structural genes to the Hypr-based chimaeras progressively reduced morbidity rates and increased animal survival times—and *vice versa* for the Hypr genes as they were progressively introduced into the Vs-based chimaeras, though the statistical support for these differences was not always significant (Fig 7A and 7B, Table 1).

**Table 1:** Morbidity and average survival time (AST) of mice, infected with control and chimaeric TBEV during the feeding of female *I*. *ricinus*.

	Morbidity,%	Significance,p =	AST, days
**Vs IC**	86	0.13**	15
**Vs[Hypr E]**	35	0.0001*	10
**Vs[Hypr PrM-E]**	75	0.55*	20
**Vs[Hypr str]**	100	0.13*	14
**Hypr IC**	100	0.13*	9
**Hypr IC[short 3`UTR]**	87,5	0.29**	>21
**Hypr[Vs E]**	75	0.1**	>21
**Hypr[Vs PrM-E]**	100	1**	20
**Hypr[Vs str]**	29	0.0001**	9

*—significance of the differences in comparison to wild-type VsIC virus was determined by single sample Student’s t-test for proportions

**—significance of the differences in comparison to wild-type HyprIC virus was determined by single sample Student’s t-test for proportions

The survival dynamics of mice infected with Vs-based chimaeras Vs[Hypr E] and Vs[Hypr prM-E] were comparable with those of wild-type VsIC and exhibited a prolonged AST of 15 and 19 days respectively. For Vs[Hypr str] the survival profile was remarkably different but closely similar to that of HyprIC virus (Fig 7A). Replacement of the E gene of VsIC with that of Hypr significantly reduced mouse morbidity from 85% to 39.5% (p < 0.01). However, when the E protein of Vs virus was expressed in the context of its authentic prM or of the entire cluster of structural genes 5'UTR-C-prM-E (i.e. Vs[Hypr prM-E] and Vs[Hypr str] respectively) morbidity was not significantly changed compared with wild-type VsIC (Fig 7A, Table 1).

Among the Hypr-based chimaeric viruses the Hypr[Vs str] was the most attenuated, showing the VsIC-like survival dynamics (Fig 7B), a low morbidity rate of 29% (p < 0.01), long survival times (increasing from 10 to > 21 days, Table 1) when compared with control HyprIC virus. In contrast, the chimaera Hypr[Vs prM-E] produced typical Hypr virus survival dynamics with 100% morbidity and a mean survival time of 9 days. Moreover, no obvious affect on mouse pathogenicity was observed due to the presence of the short 3`UTR with 99% morbidity and an AST of 9 days for HyprIC short versus 100% morbidity and an AST of 8 days for HyprIC with the long 3`UTR (Fig 7B, Table 1).

It was technically difficult to perform experimental infections in mice via the natural route, i.e. tick bite, since it was impossible to control precisely the dose of infecting virus. We assumed that variations in input dose of TBEV are mainly determined by the variations of the virus titre in female salivary glands. Therefore, to evaluate the impact of variable inoculation doses, the morbidity rate was plotted against mean virus titres that had been determined in salivary glands of infected females (SG titres) at the end of the co-feeding experiments (Fig 7C). Differences in SG titres positively correlated with differences in mouse morbidity rates (r = 0.71). However, this correlation was not strictly linear. Equivalent morbidity rates (100%) were observed following infection with HyprIC, Vs[Hypr str] or Hypr[Vs prM-E] over a variable range of SG titres (3.5–5 log10 pfu/ml). On the other hand, in the case of Vs[Hypr E] the observed reduced morbidity rate (35%) at an SG titre of 3.5 log10 pfu/ml presumably reflects the impact of genetic perturbation rather than challenge dose of virus.

### Associations between TBEV structural genes, virus replication in tick salivary glands and NVT between *I*. *ricinus* ticks

**Fig 8:**
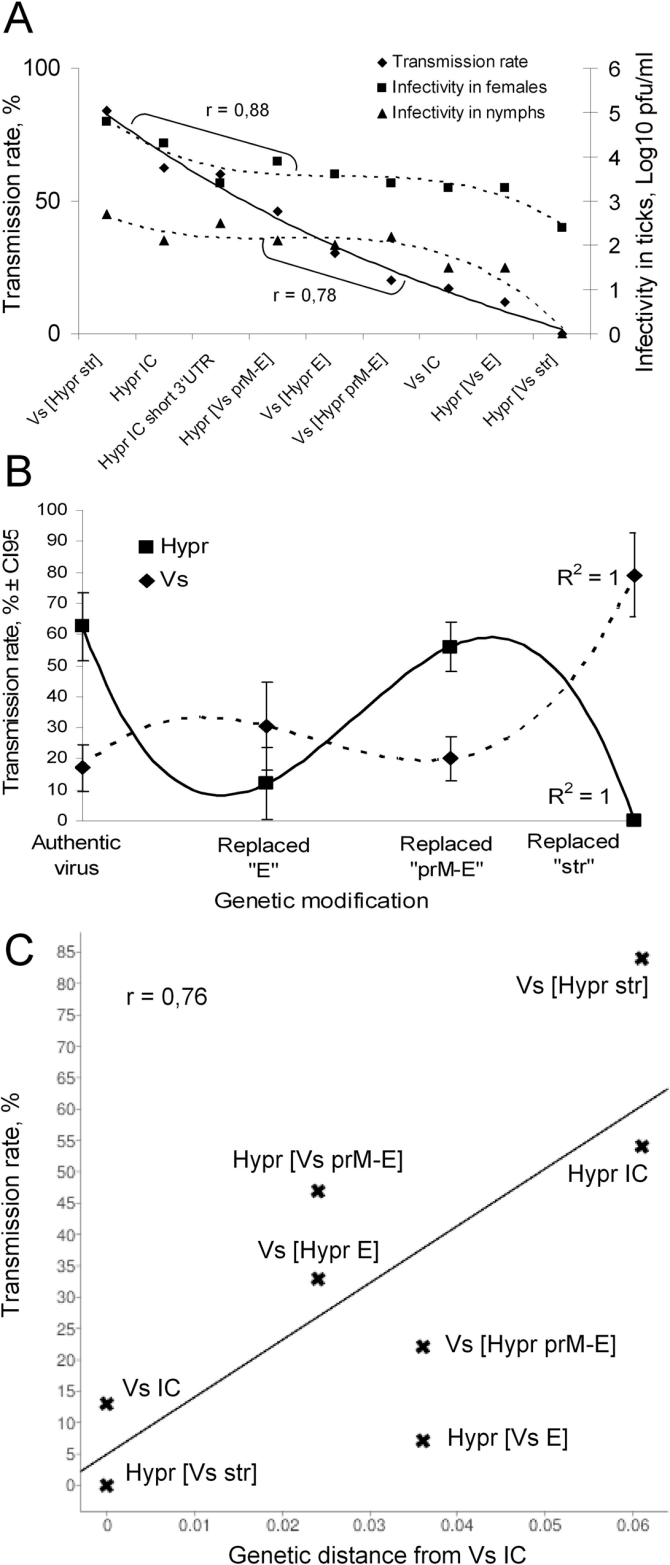
Impact of genetic background on NVT of TBEV. A) Correlation of non-viratemic transmission (NVT) rates of different TBEV chimaeras with reproduction rate in different life stages of ticks. The trend lines are estimated by the polynomial method. The Pearson`s correlation coefficients between NVT rate (solid trend line) and titres in ticks (dashed lines) are shown by brackets with indicated r values. B) Correlation of TBEV gene replacements with NVT rate. The solid and dashed trend-lines reflect the changes in Hypr and Vs NVT rate efficiency respectively, in correspondence with size of exchanged gene fragments. Trend-lines were drawn using order 3 polynomial method with R-square value shown at the right end of the corresponding trend line. C) Correlation between NVT rate and the amino acid substitutions. The amino acid differences between C-prM-E region of polyprotein of control and chimaeric viruses are plotted on the X-axis as number of amino acid substitutions per site in comparison to VsIC sequence. The NVT rates are plotted on the Y-axis. The linear regression is calculated using equation [Y = 914.53*X + 4.77] and shown as solid line (goodness of fit R^2^ = 0.58). The Pearson’s correlation coefficient (r) is shown in top left corner of the panel.

To evaluate further whether or not the observed changes in NVT rate were caused by modifications of the chimaeric viral genomes rather then titre, we applied three independent methods of regression and correlation analysis. Firstly, analysis of correlations between the NVT, titres of TBEV in salivary glands of females and in nymphs of different chimaeric viruses; secondly, regression analysis of correlations between TBEV genome fragment replacements with NVT rate and thirdly, regression analysis of correlation between NVT rate and amino acid similarity of the structural proteins of studied viruses (Fig 8A, 8B and 8C respectively). When we arranged the viruses in order of decreasing NVT rate, a strong association between TBEV NVT rate, virus titre in the salivary glands of adult ticks and nymphs and the specific TBEV genetic background was revealed. There was a strong correlation (Fig 8A) between virus titre in female ticks and NVT rate (r = 0.88), in nymphs the correlation with NVT rate was also strong, albeit less robust (r = 0.78). In summary, the pattern of change in recovered infectivity of the different viruses was very similar in nymphs and female ticks (Fig 8A) indicating equivalent decrease of virus fitness for the invertebrate host.

When relative NVT rates and corresponding genetic modifications in the VS and Hypr chimaeras were compared, exchange of homologous genes led to equivalent changes in TBEV NVT rates and increasing the size of exchanged genomic fragment, increased the impact on NVT rate (Fig 8B). Thus, replacement of E genes caused a decreased NVT rate in Hypr[Vs E] and an increased NVT rate in Vs[Hypr E] (p > 0.05). However, in the case of Hypr[Vs prM-E] versus Vs[Hypr prM-E] the changes in NVT rate were of even greater significance (p < 0.05). Moreover, when the entire structural region (including the 5’UTR) was substituted, the resultant change in NVT rate closely resembled the corresponding control viruses, i.e. either very high NVT rate in Vs[Hypr str] or complete termination of NVT rate in Hypr[Vs str] with very high significance of the observed changes (p < 0.01). The best fit statistical model to describe this process was order 3 polynomial regression (R^2^ = 1). Under this model, the differences in NVT rate of chimaeric Vs and Hypr viruses resemble sinusoidal curves which are in direct opposition. Thus, maximal NVT rate values of Vs-based chimaeric viruses correlate with minimal values of NVT rate for Hypr-based chimaeric viruses and *vice versa* (Fig 8B). As will be discussed later, these changes probably reflect the chaperone role of the prM protein and the unknown but significant role of the 5’UTR-C genetic region in the transmission process (see Discussion).

We estimated the genetic distance between the tested viruses as the number of amino acid substitutions per site in comparison with the parental VsIC sequence (only structural genes were compared). Then we plotted the amino acid distances against the NVT rate of corresponding viruses and performed the linear regression analysis. The NVT rate had a strong positive correlation with genetic distance from VsIC estimated as amino acid substitutions per site (r = 0.75, Fig 8C). The lowest NVT rate was associated with high amino acid similarity to VsIC whereas the highest NVT rate was associated with low amino acid similarity to VsIC. The control VsIC and HyprIC viruses were located at the edges of the dot-plot illustrating both largest genetic distance and fundamental differences in transmission efficiency. The chimaeric viruses with heterologous E or prM-E genes were placed in the middle region of the dot-plot indicating a restricted influence of amino acid substitutions in E and prM-E genes for NVT. However, the mutants with a heterologous ensemble of structural genes together with the 5’UTR were found in opposite corners of the dot-plot, near to the control viruses that served as donors of the 5`UTR-C-prM-E region. Thus, replacement of the ensemble of structural proteins led to the complete inversion of TBEV transmission specificity. Moreover, amino acid substitutions localized in the C protein were critically important for this process.

Thus, the correlation analyses supported the evidence that TBEV NVT rate between co-feeding ticks is largely determined by the titre the virus achieves in the salivary glands of female ticks. In conclusion, the strong correlation between the amino acid composition of the structural part of the viral genome with the NVT rate indicate that the structural proteins are the primary viral determinants for efficient NVT of TBEV between co-feeding *I*. *ricinus* ticks (Fig 8C).

## Discussion

The association of different subtypes of TBEV with particular tick species is well known and impacts on our scientific understanding of the circulation, evolution and epidemiology of TBEV in humans and wildlife species (reviewed in: [1, 21]). However, to date, there are no documented barriers to the spread of different virus subtypes between alternative species of vector ticks. Indeed there are numerous reports that following experimental inoculation any subtype of TBEV is able to replicate and be transmitted by various tick species [29, 31, 38, 39].

In this study the comparative tests of efficiency of NVT of wild-type Hypr and Vs virus (associated with *I ricinus* and *I persulcatus* respectively) revealed previously unidentified adaptive characteristics to competent tick species. Comparative analysis of a panel of chimaeric viruses supported this conclusion, revealing that in spite of the ability of divergent TBEV subtype to replicate in *I*. *ricinus*, the virus titre in female salivary glands is significantly higher for EU-TBEV and that this correlates with the rate of NVT. At the molecular level the efficiency of NVT appeared to be primarily determined by the structural gene cluster 5'UTR-C-prM-E, with a strong indication of an important contribution by the 5'UTR—C genome region. Indeed, the addition of the heterologous 5'UTR-C region to [prM-E] chimaeras was an essential requirement to increase the NVT rate of Vs[Hypr str], to values comparable with those of the control wild type HyprIC virus and in the directly opposite case, i.e. Hypr[Vs str], transmission was completely abrogated. The prM functions as a chaperone for authentic folding of the E protein; it is present in immature (intracellular) virions and is separated into its individual components, ie pr and M protein by furin-mediated cleavage during virion egress from infected cells. Consequently, mature extracellular virions contain only the M protein anchored in the cell-derived virion membrane together with the E protein [40, 41].

Under ideal circumstances similar experiments with chimaeric viruses and NVT using co-feeding *I*. *persulcatus* would have been helpful in confirming our interpretation concerning the significance of adaptation of TBEV to specific hosts and the consequences for its evolution and epidemiology. Unfortunately, despite recent advances in the establishment of a laboratory colony of *I*. *persulcatus* [42] a robust model system for this tick species is not currently available.

In contrast, efficient TBEV infection and replication in mammalian cell culture did not appear to be dependent on the structural region of the genome (with the exception of the heterologous 5`UTR-C region in the HyprIC backbone, see below), whereas cytopathic differences between Vs and Hypr were primarily determined by the non-structural protein encoding region of the virus genome. Moreover, in general chimaeras containing the 5'UTR-C from Hypr in a backbone of VsIC (Vs[Hypr str]) were generally more stable than those containing the 5`UTR-C from Vs in a backbone of HyprIC (Hypr[Vs str]). Thus, the replication dynamics in PS cells, physical stability of virions and infectivity in salivary glands of tick females for Vs[Hypr str] were comparable to controls or better. However, the reciprocal chimaera Hypr[Vs str] exhibited delayed replication dynamics in cell culture, lower thermal stability of virions and significantly decreased production of infectious particles in tick salivary glands (Figs 4, 5 and 6). Possibly, the C protein encapsidated viral genomic RNA remains associated with host cell-derived virion membranes, as has previously been described for flaviviruses [43]. Furthermore, the capsid protein is responsible for translocating the prM into the lumen of the endoplasmic reticulum whereas further cleavage of C protein by NS2B\3 protease initiates polyprotein processing and virion formation. This process is known to be highly membrane dependent. Thus, incompatibility inside the C-prM region might affect very early stages of TBEV replication or even completely abrogate the formation of infectious particles [44]. It has also been demonstrated that the genetic polymorphisms in EU-TBEV occur at a significantly lower frequency than that of SIB-TBEV or FE-TBEV [18, 27]. In this context, it seems possible that viral protease complex of Hypr virus has adapted to the less variable polyprotein sequence of the 5'UTR-C-prM region whereas the protease complex of Vs virus naturally adapted to process more diverse sequences of SIB-TBEV polyprotein. As a result, replacement of the 5'UTR-C-prM in Hypr by the Vs-derived equivalent impaired Hypr NS2B\3 protease activity, significantly reducing formation of infectious particles. In contrast, the more broadly specific Vs protease is capable of processing the heterologous 5`UTR-C-prM region of Hypr virus effectively. This assumption is also supported by the failure of the Hypr [Vs C] chimaera to produce infectious virus. All these processes (i.e., polyprotein processing, prM anchoring and capsid formation) are associated with host cell membrane structures [45], thus molecular compatibility between host cell membranes, viral non-structural and viral structural proteins appears to be a key requirement for specific host adaptation of TBEV and predetermines the effectiveness of NVT.

In spite of the fact that virus cytopatogenicity for PS cells was primarily determined by the non-structural part of the genome, the pathogenicity profile in mice depended primarely on structural genes. Most observed differences in mice reflect the impact of the genetic modifications within the TBEV genomes rather than that of virus challenge dose. The titres of chimaeras HyprIC short, Vs IC, Vs[Hypr prM-E] and Hypr[Vs E] in tick salivary glands fall within a range of 3–3.5 log10 pfu/ml and therefore differences in their pathogenicity profiles are probably caused by genetic alterations in the virus genome rather than by variability of the infecting virus dose delivered by the ticks. The significantly attenuated properties of Hypr[Vs str] in mice could be attributable to the low infectious dose (2.5 log10 pfu/ml) in ticks. However, attenuated properties of this virus were also observed in PS cells where the other viruses did not show these differences.

In conclusion, the modelling of TBEV transmission in conditions that closely resemble those in nature has revealed results previously inaccessible using *in vitro* cell culture methods. All the TBEV strains tested exhibited similar replication kinetics in cell culture but exhibited remarkable differences in their relative ability to replicate in adult ticks and nymphs. These differences ultimately determined the efficiency of NVT during tick co-feeding, a fundamental requirement for virus dissemination and survival in the natural environment. When combined with previous studies of TBEV transmission between ticks, these studies are beginning to define the underlying molecular processes that have evolved to ensure maximum virus fitness for the differing molecular machinery of distinct tick species.

## Materials and Methods

### Viruses, cells and ticks

Porcine embryo kidney cells (PS) were used to produce TBEV stocks (which were aliquoted and frozen at -70°C), to recover mutant viruses, for plaque assays and studies of cytotoxicity. Cells were maintained in RPMI 1640 media supplemented with L-glutamine, Penicillin (100 U/ml), Streptomycin (100 μg/ml) and foetal calf serum (FCS, 4%). The experimental procedures were conducted using the support media of exactly the same composition but supplemented with 2% FCS instead of 4%. The SIB-TBEV strain Vasilchenko (Vs) was isolated from the blood of a seronegative patient with fever who subsequently recovered, with no neurological sequelae [46, 47]. The prototype EU-TBEV strain Hypr was used to construct the infectious clone; it was isolated from the blood of a young patient diagnosed with TBEV in 1953 [48]. The *I*. *ricinus* ticks were bred in the Institute of Zoology, Slovak Academy of Science, Bratislava [14].

### Construction of infectious clones of Hypr virus and chimaeric Vs/Hypr viruses

Construction of the infectious clone of Vs (pGGVs) was described previously [49], [47] and will be described here as Vs IC. To construct the infectious clone of Hypr virus (Hypr IC), the RNA was extracted from 200 μl of 10% infected mouse brain suspension using Total RNA Isolation System (PROMEGA). Long high-fidelity RT-PCR was essentially performed as described in [50] using appropriate primers (available on request). The HyprIC was constructed as 2 overlapping plasmids using plasmid pBR322 (medium copy vector) and low-copy AbleK*E coli* strain (Stratagene) following the protocols [49]. The infectious clones of 9 chimaeric viruses, with genes exchanged between Vs and Hypr were constructed via a range of intermediate plasmids using the infectious clone of Vs virus [49] and HyprIC (Fig 1 and S1 Table, also described in Results). All final plasmids used to restore the full-length HyprIC and chimaeric viruses were fully sequenced using a TaqBigDye Terminator v3.1 Cycle Sequencing Kit (Applied Biosystems).

To overcome the issue of TBEV IC cDNA genome instability in *E*. *coli*, we previously developed the approach of sub-cloning single virus cDNA molecules into two overlapping plasmids that could be ligated in vitro to re-create a full-length cDNA, which acts as a template for SP6-transcription to produce synthetic full-length genomic RNA [36]. A short linker (10–15 nts) was incorporated at the boundary between the virus and bacterial sequence of the plasmid. After digestion, the linker was removed using appropriate enzymes and Sephacryl S-400, thus avoiding the isolation of long DNA molecules from the agarose gel after electrophoresis required for virus recovery [30, 36, 49]. These principles were used for the construction of the infectious clones for Hypr and also for the chimaeric viruses each of which was produced via ligation of 2 plasmids, representing structural and non-structural parts of the TBEV genome (S1 Table).

Hypr virus genome cDNA was amplified in 2 sets of long high-fidelity PCR, one representing the structural region between nucleotides 1–2461 and the second the non-structural region between nucleotides 3154–10835. These PCR fragments were cloned as two plasmids, pDGHypr1-2450 and pATHypr3154-10835. A silent BsuI site was introduced into pDGVs660-3216-BsuI at the boundary between the E and NS1 proteins (TBEV positions 2444–2450, S1 Fig) that was used for cloning of the structural part of the Hypr virus. Subsequently the plasmid pDGHypr1-2450 was extended with the Hypr-specific cDNA fragment (amplified by the RT-PCR) between positions 2450 (Bsu36I) and 3159 (ClaI) to construct pATHypr1-3159 (S1 Fig). Two final plasmids pATHypr1-3159 and pATHypr3154-10835 were ligated at a unique ClaI sitein the NS1 region, to re-create a full-length Hypr virus cDNA clone (S1 Table).

The 3`UTR of Hypr is shorter than that of some freshly isolated TBEV strains of the 3 different subtypes (Fig 2). It has been proposed that shortening the TBEV 3'UTR, by deleting the internal highly variable region occurs as the result of numerous virus passages in mice and cell culture [32, 33]. Therefore, in addition to the HyprIC with a shortened 3'UTR, the elongated 3`UTR was amplified from EU-TBEV strain IR121-122/M1 and cloned into the plasmid pATHypr3154-10835 to create a plasmid pATHypr3154-11103. The ligation of pATHypr1-3159 and pATHypr3154-11103 *in vitro* restored the full-length clone to recover Hypr virus with a long 3`UTR (Fig 1B and S1 Table).

The full-length infectious clone of the Vs pGGVs constructed previously [49] was sub-cloned into the two plasmids, pATVs1-3216 and pDGVs3211-10928 that overlap at the AvrII site, located in the NS1 protein. The virus recovered from these plasmids was named VsIC (S1 Table). The introduced Bsu36I site was used to construct 2 chimaeric viruses, one being the Vs[Hypr str] in which the structural proteins of Hypr were recombined with the non-structural proteins of the Vs virus (Fig 2 and S1 Table). The plasmids pDGHypr1-2450 and pDGVs3211-10928 were ligated in the Avr II site and the full-length clone was used to recover this virus. The second virus Hypr[Vs str], a counterpart of the Vs[Hypr str], was constructed using plasmids pATVs1-2450 and pATHypr3154-11103 (S1 Fig and S1 Table).

The silent restriction site MluI was introduced at the boundary between the M and E genes of the Vs-containing plasmid and the Hypr E gene was cloned between MluI and Bsu36I to produce plasmid pATVs660-3216 [E-Hypr]. To restore full-length virus, Vs[Hypr E], a plasmid pATVs660-3216del was constructed from the pGGVs, with the region between AgeI-AvrII being replaced with a short linker. The Vs[Hypr E] was recovered from the RNA SP6-trasncribed from plasmids pATVs660-3216 [E-Hypr] and pATVs660-3216del ligated *in vitro* in the AvrII site (Fig 2, S1 Fig and S1 Table).

Similarly the silent site XbaI was introduced at the boundary of thr M and E genes of Hypr virus. The final plasmid pATHypr1-3159[E-Vs] was ligated with pATHypr3154-11103 to construct the counterpart virus Hypr[Vs E] (Fig 2, S1 Fig and S1 Table).

Using a similar principle, the silent site BsiWI was introduced into the virus cDNA of both Vs and Hypr virus (S1 Fig) and used to construct the two counterpart chimaeric viruses with exchanged prM-E genes, designated Vs[Hypr prM-E] and Hypr[Vs prM-E] (Fig 2 and S1 Table).

### Recovery of mutant viruses

To recover each engineered virus, two plasmids with chimaeric regions of Vs and Hypr genomes that represent structural (5'UTR-C-prM-E) or non-structural (through NS1-NS5-3UTR) pregion of the TBEV genome were digested with overlapping enzymes and ligated *in vitro* to restore the full-length cDNA of TBEV as described previously [36, 49]. Each restored full-length clone was subsequently linearised by *Sma*I and used for SP6 transcription *in vitro* to produce full-length RNA. The SP6-transcribed RNA was injected intracerebrally directly into suckling mice [36, 49] or transfected into PS cells using Lipofectin reagent (Invitrogen) according to the manufacturer’s protocols. Infectious supernatant medium was collected on days 2–4 pi. The presence of virus in infected cells was confirmed by immunofluorescence microscopy using monoclonal antibodies specific for flavivirus E proteins [51] and by RT-PCR. The boundaries between Hypr and Vs genes were sequenced to confirm the identity of each chimaeric virus. Sequences of all recombinant viruses were deposited in GenBank with accession numbers KP716971-KP716978.

### Plaque assays

Aliquots of virus diluted in serum-free RPMI 1640 medium (250 μl) were added to PS cell monolayers in 24-well plates. After virus adsorption for 1 h at 37°C the inocula were removed and cells were overlaid with RPMI 1640 medium supplemented with 2% FCS and 1% SeaPlaque Agarose (Cambrex, USA). After incubation at 37°C for 5 days monolayers were fixed with 4% formaldehyde and stained with 0.05% crystal violet. Plaques were counted and virus titre was expressed as log_10_ pfu/ml.

### Cytopathic effect

Confluent monolayers of PS cells in 24-well plates were infected at least in four replicates at an estimated MOI = 1 pfu/cell. Infected cells were overlaid with 1 ml of support media and incubated at 37°C with a 5% CO_2_ atmosphere for 96 hours. The plates were fixed with 4% formaldehyde, stained with 0.05% crystal violet (CV), washed, air dried and photographed. The stain from monolayers was extracted with methanol and viable cells were quantified by measurement of optical density of extracts at wavelength 590 nm. Previously, it has been shown, that optical density of crystal violet extract is linearly related to the number of stained cells [52]. Therefore, the percentage of surviving cells in infected monolayers was quantified as a proportion of optical density of the corresponding CV extract in comparison to optical density of the CV extract from uninfected control cells (cpe = OD infected / OD control × 100%).

### TBEV reproduction dynamics in PS cells

Porcine kidney PS cells were grown overnight in 50 ml flasks to confluence and then inoculated with 0.2 ml of TBEV suspension at MOI = 1 pfu/cell. After 1 h of adsorption at 37°C, the inocula were removed; cells were washed 5 times with serum-free RPMI 1640 medium and overlaid with 5 ml of support medium containing 2% FCS. The supernatant medium from infected cells was collected at 0, 4, 8, 12, 16, 20, 24 and 72 hpi and frozen at –80°C prior to further analysis. The experiments were performed in triplicate. The titres of infectious virus at different time points were determined by plaque assay.

### Stability of TBEV virions

One millilitre of cell culture supernatant medium (CCSM) from PS cells infected with TBEV was clarified by centrifugation at 12,000 g for 10 min. Each CCSM was aliquoted (100 μl) in sterile tubes and subjected to one of the following procedures 1) frozen at -80°C (control aliquot); 2) freeze-thawed once and then frozen at -80°C; 3) freeze-thawed 6 times and then frozen at -80°C; 4) incubated at 37°C for 40°C and then at 40°C for another 40 min and then frozen at -80°C; 5) incubated at 37°C for 48 hours and then frozen at -80°C and 6) incubated at 42°C for 48 hours and then frozen at -80°C. Virus infectivity was then determined by plaque titration. Each experiment was performed in triplicate.

### TBEV co-feeding transmission between ticks

Experiments on TBEV co-feeding transmission were essentially performed as described previously [13, 14, 16, 29]. Briefly, a group of unfed *I*. *ricinus* female ticks were infected with 500 pfu of TBEV chimaeric or wild-type strain Hypr or Vs virus by inoculation into the coxal plate of the second pair of legs. Infected ticks were incubated at room temperature (24 ± 4°C) and 85–90% RH in a desiccator for 14 days. Two infected adult female ticks were allowed to feed on each of 4–8 Balb/C mice for three days simultaneously with 15 uninfected *I*. *ricinus* nymphs that were attached in close proximity to the feeding donor females. Surviving nymphs and salivary glands of donor females were used to determine the titres of infectious virus using plaque assays. The NVT rate was estimated as the proportion of nymphs that became infected. In total, 114 individual transmission experiments were taken into analysis that provided at least four independent replicates for every virus. Transmission experiments for the control viruses HyprIC long 3`UTR and VsIC H216 were also replicated in triplicate.

### TBEV pathogenicity in mouse model

Host mice after tick co-feeding were monitored during 21 days post tick bite and day of onset of symptoms of encephalitis was documented to evaluate morbidity. Mice that survived 21 days post tick bite were euthanized and haemagglutination inhibition assays were performed to detect anti-TBEV antibodies and confirm the infection. At least 8 mice were monitored for each virus and their survival probabilities and morbidity rates were calculated. The usage of animals in the experiments was approved by the State Veterinary and Food Administration of the Slovak Republic (permission numbers 12284/03-220, 2362/06-221) and by the Committee for biomedical ethics of FSPSI “Scientific Centre of Family Health and Human Reproduction Problems” (The Decision of the Committee for biomedical ethics of FSPSI “Scientific Centre of Family Health and Human Reproduction Problems” №4 from 26.10.2012). All possible efforts were taken to ameliorate animal suffering, including minimization of the number of animals used for the study, anesthesia, and euthanasia of moribund animals at the outbreak of clinical symptoms. The animals were checked twice a day in the morning and late afternoon. The general fitness of mice—based on body weight, food consumption, mobility and motion coordination were used to assess animal health, body condition, and well-being. The humane endpoint symptoms included rough fur as indicator of fever, loss of balance, and\or back limb paralysis. Consequently, the mice were euthanized the same day they showed the symptoms. No animals died without anaesthesia. Animals were euthanized by cervical dislocation under deep anaesthesia induced using carbon dioxide.

### Statistical analysis

TBEV NVT rates and infectivity are presented as mean values. Confidence intervals of 95% were estimated for each mean value and outlying variables were excluded using quartile method according to [53]. Between-group comparisons for virus titres were performed using unpaired two-tailed Student’s t-test. Between-group comparisons for NVT rates were performed using the U-criterion of Mann-Witney. Survival probabilities of infected mice were estimated using the Kaplan-Meier method. Between-group comparisons for mice morbidity rates were evaluated using single sample Student’s t-test for proportions. Values of p < 0.05 were considered as significant. The relationships between the TBEV NVT rates and virus titres in ticks were evaluated using the Pearson correlation test, the correlation was assumed to be significant at r ≥ 0.7 (p = 0.05). Data analysis was performed using SigmaPlot 11 (Systat Software Inc., USA), Statistic 6.1 and MSOffice EXCEL 2003 software.

## Supporting Information

S1 Fig Construction of chimaeric TBEV strains. Schematic representation of intermediate plasmids constructed to recover recombinant TBEV strains (Fig 1 and S1 Table). The polyproteins, with individual proteins of Hypr virus (shadowed bars) and Vs virus (white bars) are flanked with 5`and 3`UTRs (horizontal lines). Introduced silent restriction sites used for cloning are specified as stars (Bsu36I), circles (MluI), triangles (XbaI) and reverse triangles (BsiWI). The plasmid designations include authorship, virus name (Vs or Hypr) and numbers that correspond to numeration of the Hypr or Vs genome. Short linkers that were incorporated instead of deleted genes are displayed as dashed lines.
(TIF)

S1 Table Recovery of recombinant TBEV viruses. Plasmids representing structural and non-structural proteins of the TBEV regions (Fig 2) were used to re-construct the full-length infectious clone for each virus by ligation in vitro. Plasmids were designated as described in Material and Methods. Sites of ligation between plasmids for each virus are specified. Full-length infectious clones were linearized with SmaI enzyme and used for the SP6-transcription in vitro. The synthetic SP6-transcribed RNA was transfected into the PS cell using Lipofectin as described in Material and Methods.
(DOC)
