# Supplementary figures and images for: Tick-Borne Encephalitis Virus Structural Proteins Are the Primary Viral Determinants of Non-Viraemic Transmission between Ticks whereas Non-Structural Proteins Affect Cytotoxicity
Source: PLoS One. 2016 Jun 24;11(6):e0158105. doi: 10.1371/journal.pone.0158105 (PMC4920422; doi:10.1371/journal.pone.0158105)

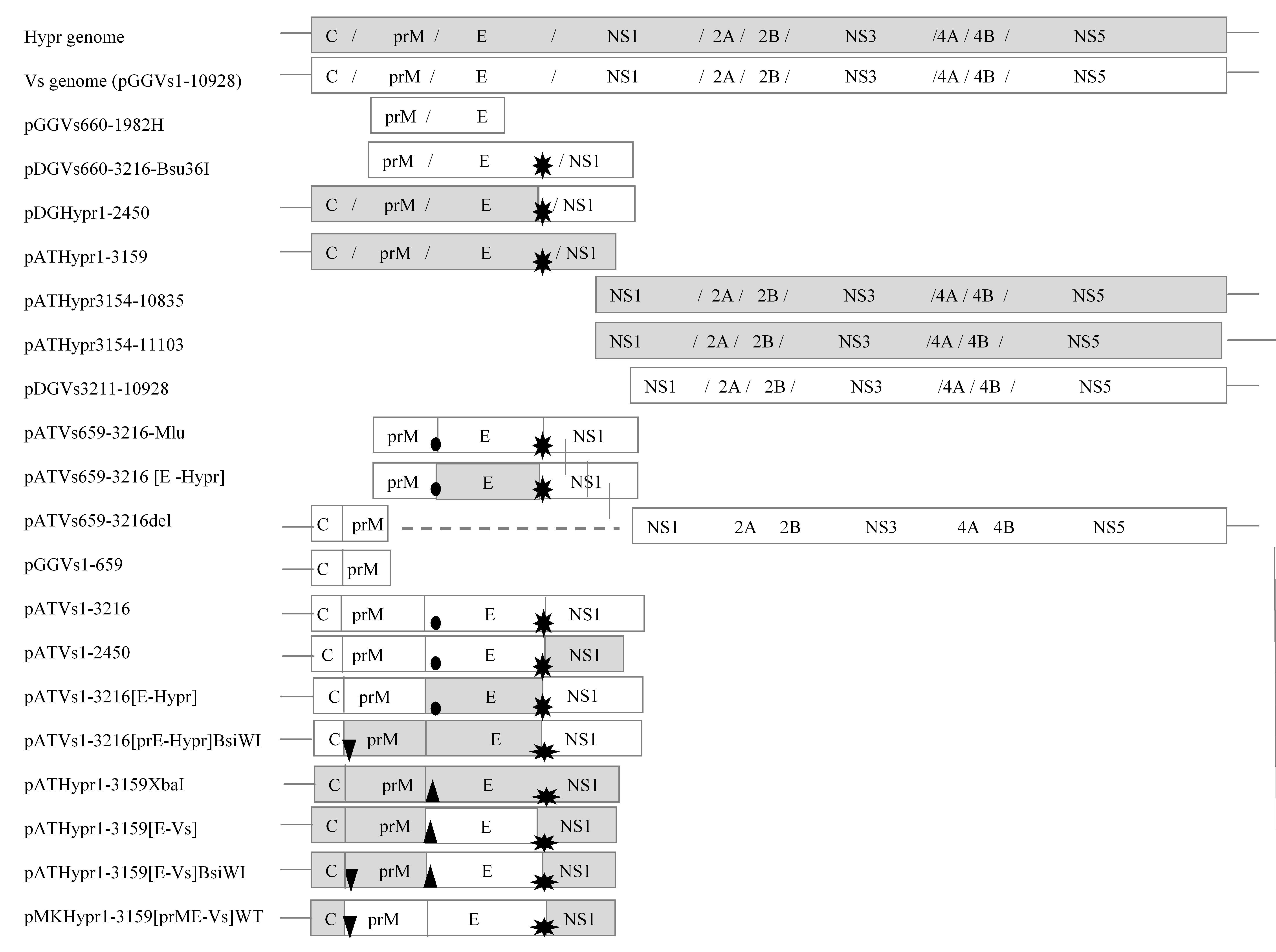

Supplement: S1 Fig — Schematic representation of intermediate plasmids constructed to recover recombinant TBEV strains (Fig 1 and S1 Table). The polyproteins, with individual proteins of Hypr virus (shadowed bars) and Vs virus (white bars) are flanked with 5`and 3`UTRs (horizontal lines). Introduced silent restriction sites used for cloning are specified as stars (Bsu36I), circles (MluI), triangles (XbaI) and reverse triangles (BsiWI). The plasmid designations include authorship, virus name (Vs or Hypr) and numbers that correspond to numeration of the Hypr or Vs genome. Short linkers that were incorporated instead of deleted genes are displayed as dashed lines. (TIF) [file pone.0158105.s001.tif]
