# Supplementary material for: Tick-Borne Encephalitis Virus Structural Proteins Are the Primary Viral Determinants of Non-Viraemic Transmission between Ticks whereas Non-Structural Proteins Affect Cytotoxicity
Source: PLoS One. 2016 Jun 24;11(6):e0158105. doi: 10.1371/journal.pone.0158105 (PMC4920422; doi:10.1371/journal.pone.0158105)
Supplement: S1 Table — Plasmids representing structural and non-structural proteins of the TBEV regions (Fig 2) were used to re-construct the full-length infectious clone for each virus by ligation in vitro. Plasmids were designated as described in Material and Methods. Sites of ligation between plasmids for each virus are specified. Full-length infectious clones were linearized with SmaI enzyme and used for the SP6-transcription in vitro. The synthetic SP6-transcribed RNA was transfected into the PS cell using Lipofectin as described in Material and Methods. (DOC) [file pone.0158105.s002.doc]

**S1 Table**. Recovery of recombinant TBEV viruses.

| Virus name | Plasmids with  structural genome region | Plasmids with  non-structural genome region | Sites for ligation |
| --- | --- | --- | --- |
| Hypr IC | pATHypr1-3159 | pATHypr3154-11103 | ClaI |
| Hypr IC short | pATHypr1-3159 | pATHypr3154-10835 | ClaI |
| Vs IC | pATVs1-3216 | pDGVs3211-10928 | AvrII |
| Vs[Hypr str] | pDGHypr1-2461 | pDGVs3211-10928 | AvrII |
| Hypr[Vs str] | pATVs1-2444Hypr2445-3159 | pATHypr3154-11103 | ClaI |
| Vs[Hypr E] | pATVs660-3216 [ Hypr E] | pATVs660-3216del | AvrII |
| Hypr [Vs E] | pATHypr1-3159[ Vs E] | pATHypr3154-11103 | ClaI |
| Vs[Hypr prME] | pATVs1-3216[Hypr prE] | pDGVs3211-10928 | AvrII |
| Hypr [Vs prME] | pMKHypr1-3159[Vs prME] | pATHypr3154-11103 | ClaI |

# Plasmids representing structural and non-structural proteins of the TBEV regions (Fig. 2) were used to re-construct the full-length infectious clone for each virus by ligation *in vitro*. Plasmids were designated as described in Material and Methods. Sites of ligation between plasmids for each virus are specified. Full-length infectious clones were linearized with SmaI enzyme and used for the SP6-transcription *in vitro*. The synthetic SP6-transcribed RNA was transfected into the PS cell using Lipofectin as described in Material and Methods.
